# Analogues of the natural product Sinefungin as potent inhibitors of EHMT1 and EHMT2

**DOI:** 10.1186/1756-8935-6-S1-P112

**Published:** 2013-04-08

**Authors:** Kanchan Devkota, Brian Lohse, Qing Liu, Ming-Wei Wang, Jens Berthelsen, Rasmus Prætorius Clausen

**Affiliations:** 1 The NNF Center for Protein Research, University of Copenhagen, Blegdamsvej 3B, DK-2200, Copenhagen, Denmark; 2 Department of Drug Design and Pharmacology, University of Copenhagen, Universitatsparken 2, DK-2100, Copenhagen, Denmark; 3 The National Center for Drug Screening and the Key Laboratory of Receptor Research, Shanghai Institute of Materia Medica, Chinese Academy of Sciences, Shanghai 201203, China

## Background

Protein Lysine methyltransferases (PKMTs) are group of histone modifiers that are responsible for the transfer of one to three methyl groups from *S*-adenosyl-L-methionine (AdoMet) to the ε-amino group of the target lysine residues in histones[1] and some non-histone targets[2]. To date more than 50 PKMTs have been identified and EHMT1 (Euchromatic Histone Methyltransferase 1, GLP, G9a like proteins) and EHMT2 (Euchromatic Histone Methyltranferase 2, G9a) are amongst the most studied ones. Genetic variations of EHMT1/2 have been associated with human diseases such as cancer, inflammatory diseases and neuro-generative disorders. As a consequence, there has been a growing interest to identify potent inhibitors of these enzymes.

## Materials and methods

We employed Sinefungin as a lead structure for the design and synthesis of a series of methyltransferase inhibitors and tested them for inhibition of EHMT1/2. The α-amino acid moiety of Sinefungin was exchanged to obtain two different series of compounds- one with the additional amino group and one without amino group. Screening of compounds were done by using a FRET-based LANCE ultra G9a histone H3-Lysine N-methyltransferase assay[3] that measures the dimethylation of a biotinylated histone H3 (1-21) peptide at lysine 9.

## Results

A series of analogues of the natural product Sinefungin was designed and synthesized, and probed for their ability to inhibit EHMT1 and EHMT2. This led to a highly potent inhibitor 4d with a K_i_ of 5 nM at EHMT1 and 24 nM at EHMT2. There was variation in the activity of the compounds and most of the compounds displayed little inhibition.

## Conclusion

Here we exchanged the α-amino acid moiety and demonstrated that it is not essential for inhibitory activity at EHMT1/2. Our results indicate that these scaffolds upon further modifications can lead to selective, potent inhibitors of EHMTs and possibly other PKMTs.
